# Prevalence of Work-Related Injury and Its Determinants among Construction Workers in Ethiopia: A Systematic Review and Meta-Analysis

**DOI:** 10.1155/2021/9954084

**Published:** 2021-07-24

**Authors:** Zemachu Ashuro, Yifokire Tefera Zele, Robel Hussen Kabthymer, Kuma Diriba, Aragaw Tesfaw, Alehegn Aderaw Alamneh

**Affiliations:** ^1^College of Health Science and Medicine, Dilla University, Dilla, Ethiopia; ^2^School of Public Health, College of Health Science, Addis Ababa University, Addis Ababa, Ethiopia; ^3^Debre Tabor University, College of Health Sciences, Debre Tabor, Ethiopia; ^4^Department of Human Nutrition and Food Science, College of Health Sciences, Debre Markos University, Debre Markos, Ethiopia

## Abstract

**Background:**

Construction is one of the highest risky jobs for accident-related fatalities and injuries globally. This systematic review and meta-analysis aimed to estimate the pooled prevalence of work-related injury and its associated factors among construction workers in Ethiopia.

**Methods:**

A systematic literature search was performed by using PubMed, Science Direct, and Google Scholar. A random-effects model was used to estimate the pooled prevalence of work-related injury and its associated factors. The heterogeneity of the studies was assessed by using the *I*^2^ test, and the presence of publication bias was evaluated by using funnel plot and Egger's test.

**Results:**

After reviewing 292 articles, we included 10 studies that fulfilled the inclusion criteria in the meta-analysis. The findings from the 10 studies showed that the pooled prevalence of work-related injury in Ethiopia was 46.78% (95% CI: 32.17, 61.38). The subgroup analysis of this study showed that the highest prevalence was reported in Addis Ababa with the prevalence of 55.9% (25.42, 86.4), followed by a study conducted in Oromia Region with a prevalence of 43.3% (33.3, 53.3). Lack of occupational safety training (OR: 2.43, 95% CI: 1.76, 3.35), not using of personal protective equipment (OR: 2.32, 95% CI: 1.80, 2.99), and male workers (OR: 2.44, 95% CI: 1.15, 5.17) were the major factors significantly associated with the occurrence of work-related injury among construction workers.

**Conclusions:**

This study confirmed that construction is still a high-risk job with a high prevalence of work-related injury in Ethiopia. The modifiable risk factors such as the use of personal protective equipment (PPE), lack of safety training, and gender were the major associated factors with injury. Therefore, a continuous safety training and awareness creation program on risk-taking behavior should be given to construction workers.

## 1. Introduction

Construction is one of the fastest growing sectors in Ethiopia where public and private sectors are investing high capital. The industrial sector showed 12.6% growth and constituted 28.1% of the total gross domestic product (GDP), and it contributed 39.5% to the overall economic expansion. The construction industry showed a 15% expansion and contributed 72.5% to the industrial output, signifying the leading role of the sector in roads, railways, dams, and residential houses construction [1, 2].

Nearly 6.5 million people work at approximately 252,000 construction sites across the nation on any day [3]. Most of the construction industry employees were less educated, none or semiskilled, untrained, and inexperienced with the tools and the hazards associated with the construction which can increase the risk of work-related injuries. The employment in the industry is mainly temporary, and once the job is over, the workers are obliged to find other jobs [4, 5].

Hundreds of millions of people throughout the world are working today under circumstances that foster ill health and/or are unsafe [6]. According to International Labor Organization (ILO), around 270 million people in the world fall victim to occupational injuries, fatal, and nonfatal every year [7–9]. OSHA's construction worker safety report showed that each year there are at least 60,000 fatal accidents on construction sites around the world. One in every six fatal accidents at work occurs in a construction site sector. Potential hazards for workers in construction include scaffold collapse, falls (from heights), electric shock and arc flash/arc blast, failure to use proper personal protective equipment, repetitive motion injuries, less regulation, and enforcement than other sectors [3, 10].

Study finding from Dessie town and Addis Ababa, Ethiopia, reported that the lowest injury prevalence of 32.6%, whereas the highest prevalence was 84.7% among construction workers, respectively [11, 12]. The wide range of prevalence of the 12-month work-related injury from the mentioned studies might be due to the lack of standard injury recording and reporting system, the difference in sample size, and the use of different data collection methods.

Though the construction sector is well known for the risk of injury, the Occupational Health Services (OHS) for workers in Ethiopia is lacking behind like in many other low and middle-income countries (LMIC) [13]. Besides, the lack of a national occupational injury recording and reporting system leads to a varied injury prevalence estimate and unable to show the magnitude of injury which further limits the prevention effort in the sector [5]. In addition, pooled data are not available on work-related injury and its determinant factors among construction workers. Therefore, this systematic review and meta-analysis was conducted to estimate the pooled magnitude of work-related injury and associated factors among construction workers in Ethiopia.

## 2. Methods and Materials

This study finding was reported based on the Preferred Reporting Items for Systematic Review and Meta-Analysis (PRISMA) statement guideline [14] (S1 File). The protocol was registered on December 07, 2020, on the PROSPERO database with the registration number CRD42020219451 and available on https://www.crd.york.ac.uk/prospero/display_record.php?ID=CRD42020219451.

### 2.1. Searching Strategies

A systematic literature search was performed between 10 November 2020 and 25 December 2020 by using PubMed, Science Direct, and Google Scholar electronic databases using key terms such as “Prevalence,” “Construction workers,” “Occupational Injury,” “Illness,” “Work-related injury,” “Construction Industry,” and “Ethiopia.” To connect, MeSH terms “AND” and “OR” were used. Searches were not restricted by publication date. We removed the duplicate articles emerging from the databases from systematic review and meta-analysis.

### 2.2. Eligibility Criteria

#### 2.2.1. Inclusion Criteria

Studies that reported the 12-month prevalence of work-related injury and its associated factors among construction workers, published and unpublished studies conducted from 2009 to 2020, and studies written within English were included during this systematic review and meta-analysis. Initially, the availability of full-text titles and abstracts of the articles was assessed. Then, the complete studies of relevant articles were reviewed.

#### 2.2.2. Exclusion Criteria

We excluded articles without full study, not fully accessed, difficult for data extraction, and outcome of interest from the analysis.

### 2.3. Data Extraction

Data from included articles were extracted using a standardized data extraction format, adapted from the Joanna Briggs Institute (JBI), by three authors (ZA, RHK, and KD) independently extracting all necessary data. The data extraction sheet include author, publication year, study area, region of the study conducted, study design, sample size, the prevalence of work-related injury, and associated factors.

### 2.4. Outcome Measurement

The outcome variable of this study was the prevalence of work-related injury and an associated factor which was defined as any physical injury resulting from construction work in the past year and estimated as the total number of workers who have injury divided by the total number of workers involved in the study multiplied by 100.

### 2.5. Data Quality Assurance

To maintain the quality of the data, we included only those studies which met the inclusion criteria under systematic review and meta-analysis.

Three reviewers (ZA, RHK, and KD) assessed the quality of included studies by using a quality assessment checklist for prevalence studies, which have nine items. Each item was classified as either low (points scored = 0) or high (points scored = 1) risk of bias. Summary of the overall risk of study bias is low risk (0–3), moderate risk (4–6), and high risk (7–9) [15]. Finally, the PRISMA 2009 checklist was used to compile the report.

### 2.6. Statistical Analysis

Data were extracted by using Microsoft Excel format and imported to STATA Version 14 and RevMan software for analysis. The pooled prevalence of work-related injury and its associated factors among construction workers were estimated by using a random-effects model. Heterogeneity among reported prevalence was assessed by using the *I*^2^ statistical test, and it can range from 0 to 100%. A value of 0% shows the absence of heterogeneity, whereas 100% indicates significant heterogeneity. The *I*^2^ statistics estimate of 0–25%, 25–50%, and 75% and above values represent low, medium, and high heterogeneities between studies, respectively [16]. The presence of publication bias was evaluated by using funnel plot and Egger's test.

## 3. Results

**Figure 1:**
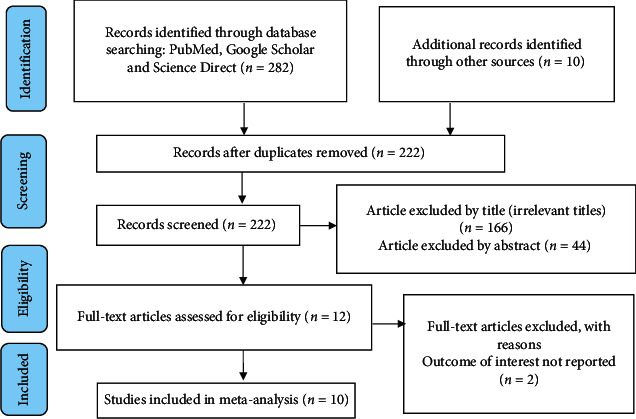
Diagrammatic flow of the data extraction process for systematic review and meta-analysis of the prevalence and determinants of work-related injury among construction workers in Ethiopia.

A total of 292 articles were identified reporting the prevalence of work-related injury and its associated factors among construction workers by using electronic databases such as PubMed, Google Scholar, Science Direct, and other sources, of which, 70 articles were removed due to duplication. From the remaining 222 articles, 166 articles were excluded by title (irrelevant titles), 44 articles were excluded by an abstract (nonrelevance), and 2 full-text articles were excluded due to the outcome of interest is not reported. Finally, 10 studies were included in systematic review and meta-analysis (Figure 1).

### 3.1. Description of Included Studies

A total of 4282 construction workers were recruited from 10 included studies conducted from 2009 to 2020. All the included studies were cross-sectional studies. The least number of participants in a study was 62 construction workers, and the largest comprised of 806 construction workers [12, 17]. The lowest prevalence (32.6%) of work-related injury was reported in the study conducted in Dessie, Amhara Region [11], whereas the highest prevalence of 84.7% was reported in a study conducted in Addis Ababa city (Table 1) [12].

### 3.2. Risk of Bias

The risk of bias for each original study was assessed by using a risk of bias assessment checklist which has nine different items [15]. Among the 10 included studies, our summary assessment showed that two studies had a moderate risk of bias [17], whereas eight studies had a low risk of bias.

### 3.3. Prevalence of Work-Related Injury

Summary of studies included showed that the lowest injury prevalence of 32.6% and the highest prevalence of 84.7% were reported among construction workers from Dessie town and Addis Ababa city, Ethiopia, respectively (Table 1) [11, 12].

### 3.4. Pooled Prevalence of Work-Related Injury

**Figure 2:**
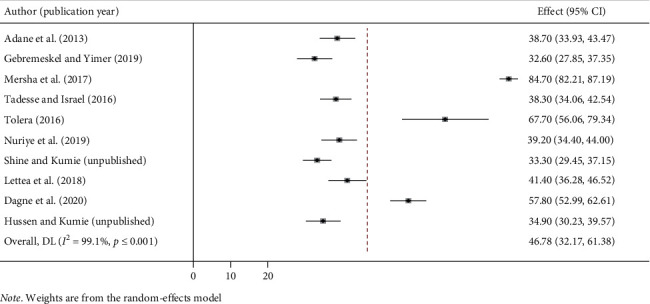
Forest plot of the pooled prevalence of work-related injury among construction workers in Ethiopia.

After reviewing 292 articles, 10 studies that fulfilled the inclusion criteria were included in the systematic review and meta-analysis. The findings from the 10 studies showed that the 12-month pooled prevalence of work-related injury among construction workers in Ethiopia by using the random-effect model was 46.78% (95% CI: 32.17, 61.38). High heterogeneity was observed across the included studies (*I*^2^ = 99.0%, *p* ≤ 0.001). From this meta-analysis, the highest prevalence was 84.7% (95% CI: 82.21, 87.19) reported in a study conducted in Addis Ababa city [12], whereas the lowest prevalence was 32.6% (95% CI: 27.85, 37.35) in Dessie town, Amhara Region (Figure 2) [11].

**Figure 3:**
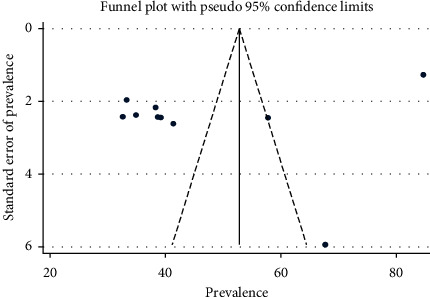
Funnel plot with 95% confidence limits of the pooled prevalence of work-related injury among construction workers in Ethiopia.

The presence of publication bias was evaluated by using funnel plot and Egger's test. The result of the funnel plot showed that there was an asymmetrical distribution of articles (Figure 3). The results of Egger's test for small-study effects showed that there was no statistically significant publication bias in estimating the prevalence of work-related injury among construction workers (*p*=0.071).

The subgroup analysis was done by the region of the country where the studies were conducted and the sample size. The highest prevalence was reported in Addis Ababa with a prevalence of 55.9% (25.42, 86.4) followed by a study conducted in Oromia Region with a prevalence of 43.3% (33.3, 53.3). The prevalence of occupational injury was higher in studies having a sample size of ≥428, 52.1% (15.97, 88.29), compared to those having a sample size of <428, 43.8% (36.30, 51.37) (Table 2).

### 3.5. Factors Associated with Work-Related Injury

#### 3.5.1. Occupational Safety Training and Work-Related Injury in Ethiopia

**Figure 4:**
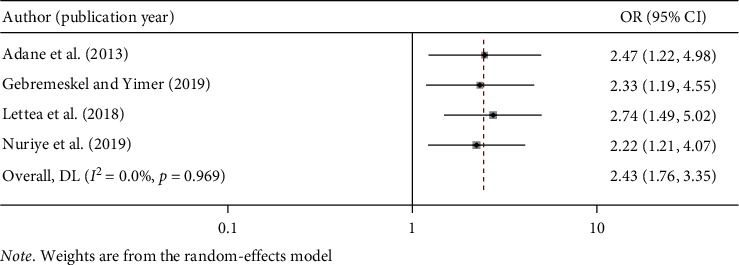
The pooled odds ratio of the association between safety training and work-related injury in Ethiopia.

The result of this meta-analysis showed that lack of occupational safety training was positively associated with occupational injury. The odd of work-related injury among workers who did not receive occupational safety training were 2.43 times more likely as compared to the odds among those who received occupational safety training (OR: 2.43, 95% CI: 1.76, 3.35). The included studies did not show heterogeneity (*I*^2^ = 0.0% and *p* < 0.989) (Figure 4).

#### 3.5.2. PPE Use and Work-Related Injury in Ethiopia

**Figure 5:**
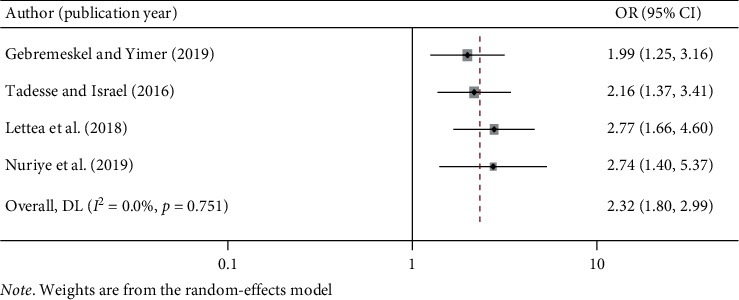
The pooled odds ratio of the association between PPE use and work-related injury in Ethiopia.

The pooled estimate of included studies showed that workers who use PPE were 2.32 times less likely to have work-related injury as compared to workers who did not use PPE (OR: 2.32, 95% CI: 1.80, 2.99). In this meta-analysis, included studies were characterized by a lack of heterogeneity (*I*^2^ = 0.0% and *p*=0.751) (Figure 5).

#### 3.5.3. Association between Gender and Work-Related Injury in Ethiopia

**Figure 6:**
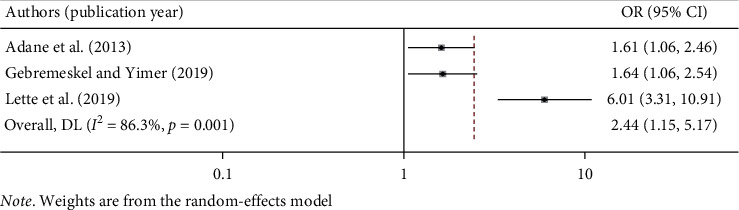
The pooled odds ratio of the association between gender and work-related injury in Ethiopia.

Work-related injuries among construction workers were significantly associated with workers' gender. Male workers were at high risk of being injured compared with female workers [11, 18, 20, 25]. The pooled result of this meta-analysis indicated that the odd of work-related injury among male workers was 2.44 times higher as compared to the odds among female workers (OR: 2.44, 95% CI: 1.15, 5.17). The included studies indicated a high degree of heterogeneity (*I*^2^ = 86.30% and *p* < 0.001); hence, random-effect meta-analysis was computed (Figure 6).

## 4. Discussion

Occupational safety and health practices are important in every organization, particularly in the construction industry. Employees in the construction industry in developing countries are frequently exposed to occupational hazards and are at risk of work-related injuries. The prevalence of occupational injuries varies from country to county. In Ethiopia, the 12-month occupational injury prevalence among construction workers ranged from 32.6% [11] to 84.7% [12].

The overall prevalence of occupational injury in Ethiopia among construction workers was 46.78%. This study result was higher than the study's findings from Iran (31%) [26] and Uganda (32.4%) [27]. The finding is in line with study from Egypt (46.2%) [28] and Ghana (57.91%) [25].

The subgroup analysis of this study indicated that the highest prevalence of occupational injury was observed in Addis Ababa city with a prevalence of 55.9%, while the lowest prevalence was observed in Amhara Region with a prevalence of 35.6%. The high prevalence of the 12-month occupational injury in Addis Ababa might be due to the lack of occupational health and safety training, lack of personal protective equipment, and extended working hours.

The present study was also aimed to identify the associated factors of occupational injury among construction workers in Ethiopia. In this study, lack of occupational safety training, lack of personal protective equipment, and gender were significantly associated with occupational injury.

The odd of work-related injury among workers who did not receive occupational safety training were 2.43 times more likely as compared to the odds among those who received occupational safety training. The finding of this study is supported by a study performed in Iran by Jazar et al. which reported that workers who attend safety training programs were 0.31 times less likely experienced work-related injury [26]. This might be due to training that provides knowledge about the presence of different safety hazards on construction and helps workers how to protect themselves. In addition, training may have an impact in changing the behaviors of workers to follow the safety precautions.

Personal protective equipment use was another factor significantly associated with the occurrence of occupational injury. Accordingly, workers engaged in the work by wearing personal protective equipment were 2.32 times less likely to develop occupational injury than those workers who did not use PPE. The finding of this study was supported by studies performed in Mumbai, India, and Kampala City, Uganda, which indicated that the use of PPE in a working environment reduces the occurrence of occupational injuries [27, 29]. This could be due to personal protective equipment protecting the worker against the hazards to which the worker may be exposed. In addition, personal protective equipment is designed to prevent occupational injury during an accident.

Furthermore, male construction workers were more vulnerable to occupational injury than female construction workers. This study showed that the odd of occupational injury among male workers was 2.44 times higher as compared to the odd of occupational injury among female workers. This study finding is consistent with a study conducted in Ghana by Amissah et al. [25], which indicated that male workers were at high risk of being injured compared with females workers, and a similar study conducted among Chinese migrant workers by Zhang et al. [30] reported that male workers are more prone to occupational injury than female workers. The possible explanation for this report is due to the difference in task and males are high in risk-taking behavior [31]. Other reasons for these differences could be that female workers used PPE more often than male workers.

### 4.1. Limitations

The limitations of this systematic review and meta-analysis is included. Studies were only written in English language and reported from two regions and one administrative town of the country due to the limited number of studies conducted among construction workers.

## 5. Conclusion

This study confirmed that construction is still a high-risk job with a high prevalence of occupational injury in Ethiopia. The modifiable risk factors such as the use of personal protective equipment, lack of safety knowledge, and gender were the major associated factors with the injury. Therefore, continuous safety training and awareness creation programs on the risk-taking behavior should be given to construction workers.

## Figures and Tables

**Table 1:** Summary of 10 studies included in the systematic review and meta-analysis of the prevalence and factors associated with work-related injury in Ethiopia, 2020.

Author	Publication year	Region	Study area	Sample size	Prevalence with 95% CI
Adane et al. [18]	2013	Amhara	Gondar town	401	38.7 (33.93, 43.47)
Gebremeskel and Yimer [11]	2019	Amhara	Dessie town	374	32.6 (27.85, 37.35)
Hanna et al. [12]	2017	Addis Ababa	Addis Ababa city	806	84.7 (82.21, 87.19)
Tadesse and Israel [19]	2016	Addis Ababa	Addis Ababa city	504	38.3 (34.06, 42.54)
Tolera [17]	2016	Addis Ababa	Addis Ababa city	62	67.7 (56.06, 79.34)
Nuriye et al. [20]	2019	Oromia	Robe town	398	39.2 (34.40, 44.00)
Shine and Kumie [21]	Unpublished	Addis Ababa	Addis Ababa city	576	33.3 (29.45, 37.15)
Lette et al. [22]	2018	Oromia	Jimma town	355	41.4 (36.28, 46.52)
Hussen et al. [23]	2019	Oromia	West Guji	405	57.8 (52.99, 62.61)
Hussen [24]	Unpublished	Oromia	Adama town	401	34.9 (30.23, 39.57)

**Table 2:** Subgroup prevalence of work-related injury among construction workers in Ethiopia, 2020 (*n* = 10).

Variables	Characteristics	Included studies	Sample size	Prevalence
By region	Amhara Region	2	775	35.6 (29.67, 41.62)
	Addis Ababa	4	1559	55.9 (25.42, 86.4)
	Oromia Region	4	1948	43.3 (33.3, 53.3)

By sample size	<428	7	2396	43.8 (36.30, 51.37)
	≥428	3	1886	52.1 (15.97, 88.29)

Overall		10	4282	46.78 (32.17, 61.38)

## Data Availability

All the datasets used to support the findings of this study are available from the corresponding author upon request.

## Abbreviations

ILO: International Labor Organization
LMIC: Low and middle-income countries
PPE: Personal protective equipment
OHS: Occupational health services
OR: Odds ratio.
